# Oscillating focus of SopA associated with filamentous structure guides partitioning of F plasmid

**DOI:** 10.1111/j.1365-2958.2007.05728.x

**Published:** 2007-06-01

**Authors:** Toshiyuki Hatano, Yoshiharu Yamaichi, Hironori Niki

**Affiliations:** 1 Microbial Genetics Laboratory, Genetic Strains Research Center, National Institute of Genetics 1111 Yata, Mishima, Shizuoka 411-8540, Japan.; 2 Department of Genetics, SOKENDAI 1111 Yata, Mishima Shizuoka 411-8540, Japan.

## Abstract

The F plasmid is actively partitioned to daughter cells by the *sopABC* gene. To elucidate the partitioning mechanisms, we simultaneously analysed movements of the plasmid and the SopA ATPase in single living cells. SopA, which is a putative motor protein assembled densely near nucleoid borders and formed a single discrete focus associated with less dense filamentous distribution along the long axis of the cell. The dense SopA focus oscillates between cell poles. The direction of the plasmid motion switches as the SopA focus switches its position. The velocity of the plasmid motion stays constant while it oscillates moving towards the SopA focus. The low density filamentous distribution of SopA persisted throughout the SopA oscillation. The focus associated with filamentous distribution of SopA was also observed in a cell without nucleoid. The SopA filament may guide the movement of the plasmid as a railway track and lead it to cell quarters.

## Introduction

In bacteria, extra-chromosomal elements have mechanisms that maintain them stably in their host cells. These mechanisms include regulation of replication, physical separation of replicated copies and partitioning of copies to daughter cells (Hiraga, 2000; Gordon and Wright, 2000; Draper and Gober, 2002). Partitioning is crucial for low copy number plasmids. The partitioning mechanism of many plasmids is governed by a set of three genes (Gerdes *et al*., 2004); one is *cis*-acting, and functions as a centromere-like site. The other genes encode an ATPase and a DNA binding protein that binds to the centromeric site respectively. In the case of the *sopABC* mechanism of F plasmid, a centromeric site of the F plasmid, *sopC*, is specifically bound by SopB (Mori *et al*., 1989).

The SopA ATPase interacts with the SopB–*sopC* complex that the SopB protein binds to centromeric site *sopC*. Subcellular localization of actively partitioning F plasmid has been visualized by fluorescence *in situ* hybridization (FISH) (Niki and Hiraga, 1997) and green fluorescent protein (GFP)-tagging methods (Gordon *et al*., 1997). F plasmids that are actively segregated by the *sopABC* partitioning genes are localized at specific subcellular sites during the *E. coli* cell division cycle. F plasmid is primarily located at midcell in newborn cells. After replication of the plasmid, each daughter plasmid migrates towards the 1/4 and 3/4 positions of cell length. Separated F plasmids are tethered at the cell quarters until the host cell divides into two daughter cells. Using immunofluorescence microscopy, discrete fluorescent foci of SopB in cells are observed in the presence of the *sopC* DNA segment (Hirano *et al*., 1998), and SopB-CFP also forms fluorescent foci in a living cell (Lim *et al*., 2005). As SopB binds to *sopC* in the actively partitioning plasmid, the discrete fluorescent foci of SopB are formed on the plasmid DNA. Even if SopA is defective, the SopB–*sopC* complex can be localized at midcell, but not the 1/4 and 3/4 positions of cell length (Yamaichi and Niki, 2004). ATP hydrolysis of SopA is required for F-plasmid partitioning (Libante *et al*., 2001). Thus, SopA is essential for faithful movement of the plasmid from midcell to the cell quarters.

Many homologues of SopA have been found in bacteria, and SopA belongs to a large family of partition-related proteins that also includes MinD, a cell division site regulator (Yamaichi and Niki, 2000). Spatial oscillation of MinD (Raskin and de Boer, 1999) and some of the homologues of SopA (Marston and Errington, 1999; Quisel *et al*., 1999; Ebersbach and Gerdes, 2001) have been demonstrated, and these proteins have been observed to form a helical shaped structure in the cell (Shih *et al*., 2003; Ebersbach and Gerdes, 2004). Oscillation of SopA was also observed under the induction of SopA-GFP expression, and SopA oscillates inside live cells with a period of 20 min (Lim *et al*., 2005). In addition, it is reported that helix-like structures of SopA were observed in despite low resolved images under an immunofluorescence microscopy (Adachi *et al*., 2006). A mutated SopA is fused with GFP, and this mutant SopA-GFP forms long filaments in the absence of the other *sop* genes (Lim *et al*., 2005). Furthermore, SopA has been shown to form filaments *in vitro* in an ATP and SopB-dependent manner (Lim *et al*., 2005; Bouet *et al*., 2007).

SopA is a putative motor protein for movement of the plasmid from midcell to the cell quarters, although it is not clear how the ATPase generates the driving force for plasmid segregation. Based on the properties of the ATPase, the partitioning machinery for plasmids can be divided into two families: the actin-type ATPase family, and the Walker-type ATPase family (Gerdes *et al*., 2000). The ATPases play a critical role in migration of plasmids towards daughter cells, and thus presumably function as motor proteins to generate a driving force for the bipolar movement. For example, ParM, which is encoded on a R1 drug-resistance plasmid, is an actin-type ATPase and considered to be a prokaryotic actin homologue (van den Ent *et al*., 2002). *In vivo* and *in vitro*, ParM forms a filamentous structure with the plasmids at both ends, and then pushes the plasmids towards the tips of the cell pole (Moller-Jensen *et al*., 2003; Garner *et al*., 2004). In contrast, the Walker-type ATPases can be found encoded on P1 phages and F plasmids as ParA and SopA respectively. The ATPase activity of SopA is about 1000-times weaker than that of the typical Walker type ATPase (Watanabe *et al*., 1992; Libante *et al*., 2001). The ATPase activity is stimulated in the presence of DNA, and further by addition of the SopB protein. Interestingly, the SopB protein alone fails to stimulate the ATPase activity of SopA (Watanabe *et al*., 1992). It is still unknown that how ATP hydrolysis by the SopA ATPase contributes to the plasmid segregation.

Although SopA is polymerized *in vitro* in the presence of SopB and *sopC* containing DNA (Lim *et al*., 2005), DNA itself inhibits polymerization of SopA (Bouet *et al*., 2007). SopB counteracts this DNA-mediated inhibition by masking DNA. SopB spreads from *sopC* (Biek and Strings, 1995; Lynch and Wang, 1995; Rodionov *et al*., 1999) and masks plasmid DNA. This allows SopA polymerization at plasmid DNA. In fact, SopA colocalizes with SopB (Lim *et al*., 2005; Adachi *et al*., 2006). Thus, it is thought that SopA mediate plasmid separation via its polymerization or extension between two daughter plasmids including the SopB–*sopC* complex (Lim *et al*., 2005). However, it is not certain that the wild-type SopA forms a filament that can extend by polymerization in a living cell. On the other hand, theoretical model is proposed to account to the *parABS* (Hunding *et al*., 2003) and the *sopABC* partitioning mechanism (Adachi *et al*., 2006). Based on reaction-diffusion equations, the models attempt to explain bipolar migration of plasmid DNA. Some aspects of the plasmid partitioning mechanisms are consistent with the models. Further experiments are needed to verify the models properly.

Here we simultaneously analysed movement of SopA and plasmid DNA in a living cell. Active form of SopA-GFP or YFP made it possible to address some crucial questions; Does oscillating SopA form a filamentous structure in the cell? If so, is the oscillation of SopA coupled to the bipolar movement of plasmid DNA? This is the first attempt to analyse the spatial dynamic relationship between SopA and the SopB–*sopC* complex of the F plasmid. Our study provides new clues as to how the putative SopA motor protein contributes to plasmid partitioning.

## Results

### Labelling of SopA and partitioning plasmid DNA

**Fig. 1:**
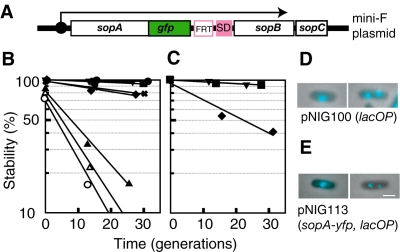
Structure, stability and subcellular localization of mini-F plasmids A. Schematic representation of the structure of the mini-F plasmid carrying the *sopA-gfp* fusion gene (pNIG105). The *gfp* gene replaced the stop codon of the *sopA* gene and the Shine-Dalgarno (SD) sequence was inserted upstream of the *sopB* gene by homologous recombination. FRT; FLP recombinase Recognition Target. B and C. TH456 cells harbouring pXX325 (*sopA*^+^*B*^+^*C*^+^ closed circle), pXX327 [Δ(*sopABC*); closed triangle], pNIG152 (Δ*sopA, sop*^+^*B*^+^*C*^+^ open triangle), pNIG138 (Δ*sopA, sop*^+^*B*^+^*C*^+^*, lacO*; open circle), pNIG105 (*sopA-gfp, sopB*^+^*C*^+^ cross), pNIG100 (*sopA*^+^*B*^+^*C*^+^*, lacO*; inverted triangle), pNIG111 (*sopA-yfp, sopB*^+^*C*^+^ square), or pNIG113 (*sopA-yfp, sopB*^+^*C*^+^, *lacO*; diamond) were grown in L medium with 20 μg ml^−1^ ampicillin at 37°C and then transferred to L medium without ampicillin with (C) or without (B) 0.2% arabinose added to the media. The time of transfer was considered as time = 0. D and E. Combined images of the phase-contrast and the LacI-CFP fluorescence micrographs. TH456 cells harbouring pNIG100 (*sopA*^+^*B*^+^*C*^+^*, lacO*, D) or pNIG113 (*sopA-yfp, sopB*^+^*C*^+^, *lacO*, E). Cells were exponentially grown at 37°C in M9 succinate medium with supplements, and synthesis of the LacI-CFP fusion protein was induced by addition of 0.2% arabinose to the media. Images are shown of one focus of LacI-CFP localized at midcell, and two foci of LacI-CFP localized at the cell quarter positions. Bar indicates 1 μm.

To investigate the behaviour of the putative motor protein, SopA, we attempted to observe the dynamics of subcellular localization of SopA in a living cell. We constructed SopA proteins fused to a green or yellow fluorescent proteins (SopA-GFP or SopA-YFP respectively), and the *sopA* gene was replaced with one of the fusion genes on the F plasmid (Fig. 1A and Fig. S1). Thus, expression of both the fused *sopA* and *sopB* genes was controlled by the native regulatory region of the *sopAB* operon. Expression level of SopA-YFP or SopA-GFP fusion proteins (70.7 kDa) was confirmed by Western blotting measurement using anti-SopA or anti-GFP or YFP antibody (Fig. S2A and C). Quantification analysis using the blotted membrane treated with anti-SopA antibody demonstrated that the expression level of SopA-GFP or SopA-YFP increased less than twofold or threefold that of the SopA protein, respectively (Fig. S2B). This increase of SopA-GFP or SopA-YFP did not affect to the active partitioning of F plasmids as showed in Fig. 1B.

To assess the biological function of the fusion proteins, we measured the stability of the plasmids in host cells (Fig. 1B). Plasmids that encoded *sopA-yfp* (pNIG111) were as stably maintained in host cells as the wild-type plasmid pXX325. Although the stability of plasmids that encoded *sopA-gfp* (pNIG105) was slightly decreased relative to pXX325 and pNIG111, pNIG105 was much more stably maintained in host cells than pNIG152, which lacks *sopA* and pXX327, which completely had lost the active partitioning mechanism (Fig. 1B). Therefore, SopA-GFP or SopA-YFP retains the biological function sufficiently to carry out active plasmid partitioning in the presence of SopB and *sopC*.

To label the partitioning plasmid, DNA fragments containing the binding sites for the LacI repressor were embedded in the plasmids and the LacI repressor protein fused to a cyan fluorescent protein (CFP) was expressed under the control of arabinose inducible promoter. The C-terminal 11 amino acid residues of the LacI protein were deleted to avoid tetramerization (Straight *et al*., 1996). As even moderate expression of LacI-CFP for visualization of the LacI biding site has been reported to affect cell growth when the binding site was embedded in chromosome near replication origin, *oriC* (Lau *et al*., 2003), we reduced the induction level of LacI-CFP by inserting the *lacI-cfp* gene in the chromosome instead of in a multicopy plasmid. Even in the presence of arabinose as an inducer, expression of LacI-CFP from the single copy gene on the chromosome did not affect the colony formation of cells in which the LacI-CFP binding site was embedded in the chromosome near *oriC* (data not shown). We measured the stability of the plasmid containing the lacI binding sequence (pNIG100) in this host cell (Fig. 1B). The plasmid pNIG100 was stably maintained over 12 h incubation (about 27 generations). When the host cells were cultivated in medium including arabinose, the stability of pNIG100 was not at all affected by the expression of LacI-CFP (Fig. 1C). Under these culture conditions, the induced LacI-CFP protein formed clear fluorescent foci at the cell quarters in the cell as it binds to the plasmid DNA (Fig. 1D). Thus, binding of LacI-CFP protein to the plasmid was judged not to interfere with the SopABC partitioning mechanism.

To track the fluorescent foci of SopA-YFP and LacI-CFP simultaneously, we constructed a plasmid that was hybrid of pNIG111 and pNIG100, pNIG113, which encoded both *sopA-yfp* and the *lacO* array. The stability of pNIG113 was slightly decreased relative to pNIG100 and PNIG111, and induction of LacI-CFP by the addition of arabinose reduced its stability still further (Fig. 1B and C). Nevertheless, the stability of pNIG113 was much higher than pNIG138, which lacks *sopA* harbouring *lacO array* and pXX327, which lacks the *sopABC* genes, indicating that the SopABC partitioning mechanism functioned sufficiently well for this study. Thus, LacI-CFP also formed cellar fluorescent foci at the cell quarters in the cell (Fig. 1E).

### Localization of plasmid DNA near the cell quarters

**Fig. 2:**
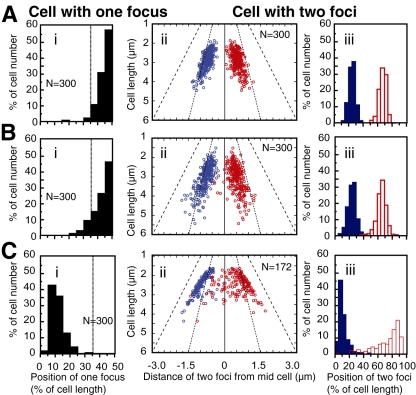
Subcellular localization of mini-F plasmids. The mini-F plasmids were detected by fluorescence of the LacI-CFP fusion protein bound to the *lacO* array, and the positions of the LacI-CFP foci were measured in cells with one or two foci. TH456 cells harbouring the mini-F plasmids were grown to log phase in M9 succinate medium with supplements with 0.2% arabinose at 37°C. A. pNIG100 (*sopA*^+^*B*^+^*C*^+^, *lacO*). B. pNIG113 (*sopA-yfp*, *sopB*^+^*C*^+^, *lacO*). C. pNIG101 [Δ(*sopABC*), *lacO*]. Cells with a single focus were statistically analysed and the distribution frequencies of the foci are shown in the histograms (i). The position of a focus is shown as a percentage of cell length. The dotted line indicates a position 35% along the cell length. Cells with two fluorescent foci were statistically analysed (ii). The distances of LacI-CFP foci from the midcell are plotted versus cell length. For each pair of foci from a cell, the focus closest to the cell pole is shown in blue. The distance of the other focus in the same cell from the same pole is also shown (red). Dotted lines indicate the 1/4 and 3/4 positions along the cell length, dashed lines indicate the position of cell poles, and solid lines indicate the midcell position. The histograms show the distribution frequencies of the foci (iii).

Previous reports indicate that the actively partitioning F plasmid is located at the cell quarters (Gordon *et al*., 1997; Niki and Hiraga, 1997). The single plasmid is mainly located at midcell, and after replication the plasmids migrate to positions 1/4 and 3/4 along the cell length. We determined the subcellular localization of newly constructed plasmids pNIG100 and pNIG113 by using the fluorescence foci of LacI-CFP, and obtained the similar results regarding the subcellular localization of the two active partitioning plasmids (Fig. 2A and B). Although the distribution of a single focus of pNIG113 (Fig. 2B, i) was a little broader than that of pNIG100 (Fig. 2A, i) and a small fraction of pNIG113 was localized at a position 20–35% along the cell length, pNIG100 and pNIG113 were both mainly located near midcell (at positions 35–50% position along the cell length). When two foci were present in a cell, they were localized at/near positions 1/4 and 3/4 along the cell length (Fig. 2A, ii, iii and Fig. 2B, ii, iii). The *sopABC* deletion mutant plasmid (pNIG101) was unstable and the distributions of the plasmids were different from others (Fig. 2C). These results indicated that daughter copies of the plasmids migrated to the cell quarters prior to segregation into the daughter cells.

### Assembly of SopA focus at the nucleoid tip

**Fig. 3:**
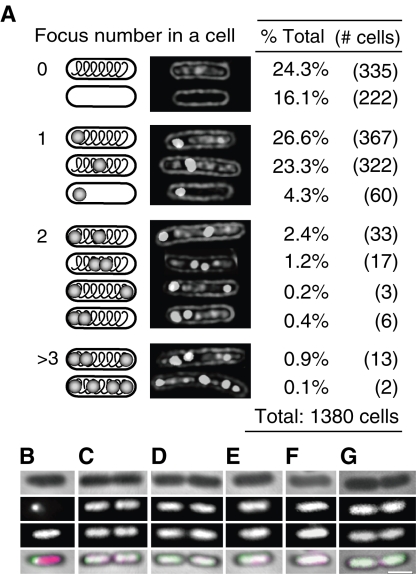
Analysis of SopA localization. A. TH456 cells carrying pNIG105 were grown to log phase at 30°C in M9 succinate medium with supplements, and embedded in an agar medium to allow images of SopA-GFP to be captured. Cell membranes were stained with FM4-64. Sectional images were processed by using a deconvolution algorithm. Merged images showing SopA-GFP and the cell membrane are shown. Cells (*n* = 1380) were categorized according to the number of SopA-GFP foci and the presence of the filaments, and the types of the cells are represented as diagrams. B–G. Localization of SopA-YFP in cells with F plasmids with various mutations. TH456 cells carrying the mini-F plasmids were exponentially grown at 30°C in M9 succinate medium with supplements, and then 1 μg ml^−1^ Hoechst 33342 was added to the medium for 1 h. B. pNIG111 (*sopA-yfp*, *sopB*^+^*C*^+^). C. pNIG120 (*sopA-yfp*, Δ*sopB*, *sopC*^+^). D. pNIG121 (*sopA-yfp*, *sopB*^+^, Δ*sopC*). E. pNIG122 [*sopA-yfp*, Δ(*sopBC*)]. F. pNIG140 (*sopA*^K120R^-*yfp*, *sopB*^+^*C*^+^) G. pNIG141 (*sopA*^K120Q^-*yfp*, *sopB*^+^*C*^+^). From top to bottom rows, the images are phase-contrast, SopA-YFP and DNA images, and merged images of phase-contrast, SopA-YFP (green) and DNA (magenta). Bar indicates 1 μm.

To investigate the distribution dynamics of the SopA protein, we observed the subcellular localization of the SopA-GFP and SopA-YFP in living cells. We found that the SopA protein tended to form a single fluorescent focus in majority (54.2%) of the cells (Fig. 3A). On the other hand, only 5.2% of cells had multiple foci. Because most cells had a single focus throughout the cell division cycle, only one of daughter cells could receive the focus and the other could not (Fig. S3B). In fact, 40.4% of cells had no strong SopA-GFP focus, although the mini-F plasmid encoding the *sopA-gfp* gene was stably maintained in much more than 40% of cells in the population (Fig. 1B).

The SopA foci were located near the cell poles, but not at their tips. This positioning pattern was similar to that of replication *oriC* and replication terminus that were localized at the nucleoid border during the cell division cycle (Niki and Hiraga, 1998). To confirm the subcellular positioning of SopA, cells were stained using DNA-specific fluorescent dye (Hoechst33342). The SopA-YFP foci were indeed located at the tip of nucleoid (Fig. 3B and Fig. S3A). Thus, the focus of SopA-YFP was not localized at midcell where the plasmid DNA could be localized (Fig. S3C). Further analysis of SopA-GFP and SopA-YFP fluorescence revealed that in addition to the strong focus described above, a small fraction of the labelled SopA proteins formed a filamentous structure in cells (see below).

### Requirement for ATPase activity, SopB and sopC for the assembly of SopA focus

In order to eliminate the possibility that fusion of the fluorescent protein to SopA caused artificial aggregation of the fusion proteins inside the cells to form the observed foci, we analysed the localization of SopA-YFP by combinations with plasmids with various mutations of the *sopABC* genes. For the *sopB* deletion plasmids, SopA-YFP was distributed over the whole nucleoid in cells (Fig. 3C). Similar results were obtained using deletion mutants for *sopC* and both *sopB* and *sopC* (Fig. 3D and E). It has previously been shown that focus formation of SopA-GFP required all three components including the wild-type SopA, SopB and sopC DNA (Lim *et al*., 2005).

To test whether formation of the SopA-YFP foci depended on the ATPase activity of SopA, a point mutation was introduced into the Walker motif A of the *sopA-yfp* gene, and subcellular localization of the mutated fusion protein was analysed. We constructed two types of the mutated fusion genes: first, SopA^K120R^ mutated protein, which has lost the ATPase activity and second, SopA^K120Q^ mutated protein, which retains almost the same ATPase activity as the wild type, but its activity is not stimulated by SopB (Libante *et al*., 2001), whereas the ATPase activity of the wild-type SopA is enhanced by SopB (Watanabe *et al*., 1992). Both of the mutant SopA fusion proteins were distributed over the whole nucleoid, and did not assemble as discrete fluorescent focus (Fig. 3F and G). These results suggest that the SopA focus formation observed with the fusion protein reflects the functional behaviour of SopA for plasmid partition.

### Oscillating SopA, and followed by plasmid DNA

**Fig. 4:**
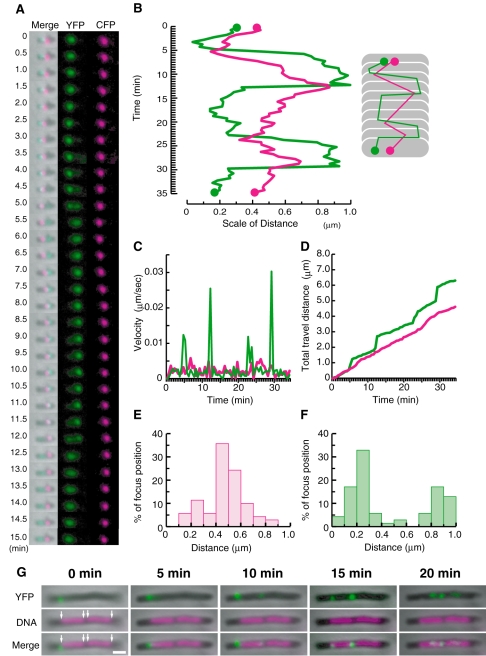
Analysis of movement of SopA and plasmid DNA A. Time-lapse images of SopA-YFP and LacI-CFP (plasmid DNA). TH130 cells carrying pNIG113 (*sopA-yfp*, *sopB*^+^*C*^+^, *lacO*) were grown to log phase at 30°C in M9 succinate medium with supplements. Images were captured for 35 min with intervals of 30 s, and each frame taken during the first 15 min is shown. Merged images showing phase contrast images, fluorescence images of SopA-YFP (YFP), and fluorescence images of LacI-CFP (CFP) are also shown (Merge). B. Kymographs of movement of SopA-YFP and LacI-CFP. To analyse the movement of SopA foci, the brightest focus was automatically selected in each cell. The point with the highest intensity for each fluorescent focus was traced, and a track is shown that represents movement of the foci in a cell. Scale bar indicates 1 μm, as the distance of migration. The diagram represents movement of SopA-YFP (green) and LacI-CFP (magenta). C. Velocity of movement of SopA-YFP (green) and LacI-CFP (magenta). D. Total travel distance of SopA-YFP (green) and LacI-CFP (magenta). E and F. Average position of SopA-YFP (F) and LacI-CFP (E). G. Time lapse images of SopA-YFP (green) and the nucleoid (magenta) in a cell treated with cephalexin (10 μg ml^−1^). TH456 cells carrying pNIG111 (*sopA-yfp*, *sopB*^+^*C*^+^) were grown to log phase, and cephalexin (10 μg ml^−1^) was added to the media for 2 h. A cell harbouring two elongated nucleoids is shown. Arrows indicate nucleoid tips. Images were captured for 20 min with intervals of 5 min. Bar indicates 1 μm.

Fluorescent foci for SopA-YFP and LacI-CFP were simultaneously observed in a LacI-CFP-expressing living cell that, and time-lapse observations were performed to analyse the subcellular localization dynamics of both types of foci during plasmid segregation. The fluorescence of LacI-CFP was very faint compared with that of SopA-GFP or SopA-YFP, so the length of time for which we could simultaneously observe plasmid DNA and SopA was restricted to less than 20 min. Figure 4 shows the movement of the plasmid DNA and SopA over 35 min, because for this cell the conditions for detecting fluorescence signals were better than for others. When a single fluorescent focus of SopA-YFP was observed, it changed location from one cell pole to the other (Fig. 4A). We observed that the SopA-YFP focus near one pole gradually disappeared within a few minutes, after which a new focus appeared near the opposite pole (Fig. 4A). The exposure time is less than 1 s, which is much shorter than intervals (30 s). It might not be the case that movement of a focus along a track during the exposure. Thus, the SopA-YFP focus did not migrate towards the opposite pole directly, but SopA-YFP near one pole dispersed within the cell over a short period and then reassembled around the opposite pole to produce a new fluorescence focus.

The single fluorescent focus of SopA-YFP was located near the cell poles, but not at their tips. The exact location of the focus was at the tip of the nucleoid (see above). In contrast, the fluorescent focus of LacI-CFP at the plasmid DNA was continuously detected in the cell during oscillation of the SopA-YFP focus and it moved towards the SopA focus (Fig. 4A). Other cells showed the similar results of SopA and mini-F movement, although time of their observation was shorter than that of Fig. 4A. These results indicate that the periodical disassembly and assembly of SopA-YFP was somehow tied to the plasmid movement.

Movement of both of the fluorescent foci, SopA-YFP and LacI-CFP in the cell was illustrated in a kymograph, which visualized the traces of centre of the fluorescent foci in a series of time-lapse pictures (Fig. 4B, and Fig. S4A). The SopA-YFP focus moved backwards and forwards along long axis of the cell length four times during a 35 min time-lapse observation. Oscillation of SopA was similar to that of the Min proteins, except for the interval of oscillation. The interval of oscillation of Min proteins is less than 1 min (Raskin and de Boer, 1999). In contrast, the intervals of oscillation of the SopA foci varied from a few minutes to more than 10 min in each cell. A single fluorescence focus of LacI-CFP chased after the SopA-YFP focus, and then LacI-CFP also oscillated along the long axis of the cell. The SopA-YFP focus started to disperse when the LacI-CFP focus came near it. After reassembly of SopA-YFP focus at the opposite pole, the direction of movement of LacI-CFP reversed immediately, and the focus continued to follow that of SopA-YFP, and thus the focus of LacI-CFP did not linger at the poles. The kymograph also could allow estimating velocity of the foci. During the ‘chase’, the velocity of LacI-CFP was almost constant and did not accelerate in response to the rapid movement of the SopA-YFP focus (Fig. 4C). While the cumulative travel distance of LacI-CFP was proportional to the time of the movement, the cumulative travel distance of SopA-YFP focus was higher and the difference reflected its sudden increase in velocity of the SopA movement towards the opposite pole (Fig. 4D). In all our analyses, the focus of SopA-YFP started to migrate towards opposite pole before plasmid DNA did. Thus, the oscillation of the SopA-YFP affected the directional movement of the plasmid DNA without changing its velocity.

When either *sopB* or *sopC,* or both were deleted from the F plasmid, or the SopA ATPase was inactivated, the SopA-YFP focus was never observed to oscillate, but instead was found to be evenly distributed over the whole nucleoid (Fig. 3C–F). Thus, SopB and *sopC*, as well as the ATPase activity of SopA are all required for the oscillation of SopA-YFP.

### Wandering plasmid DNA near midcell

Previous works have shown that the mini-F plasmid is primarily localized at midcell (Gordon *et al*., 1997; Niki and Hiraga, 1997), suggesting that the plasmid could be fixed there. The results of the present study, as shown in Fig. 2, were consistent with this notion. However, our time-lapse observations of living cells revealed that the plasmid DNA actually migrated backwards and forwards along long axis of the cell length. Although these observations may appear contradictory, the average location of the oscillating plasmid observed in the time-lapse experiment was near the midcell (Fig. 4E), which is consistent with the previous reports that showed plasmids were localized at mid cell. On the other hand, the average location of the SopA-YFP focus was at the two poles (Fig. 4F). Thus, the mini-F plasmid remains near the midcell in living cells, but it is not anchored at a specific site constantly.

### Oscillation of SopA between the nucleoid tips

The foci of SopA-YFP and SopA-GFP were located at the nucleoid tip (Fig. 3A, and Fig. S3A), and thus we hypothesized that oscillation of SopA-YFP probably occurred between the two tips of the nucleoid. To test this idea, oscillation of SopA-YFP was observed in a cell in which cell division was inhibited by addition of cephalexin to produce elongated cells with elongated nucleoids. In the elongated cells shown in Fig. 4G, two elongated nucleoids were formed and the focus of SopA-YFP oscillated from a tip of one nucleoid to the other tip. This result supports the idea that the SopA-YFP focus is oscillated between nucleoid tips.

### After separation, one plasmid stays and the other goes

**Fig. 5:**
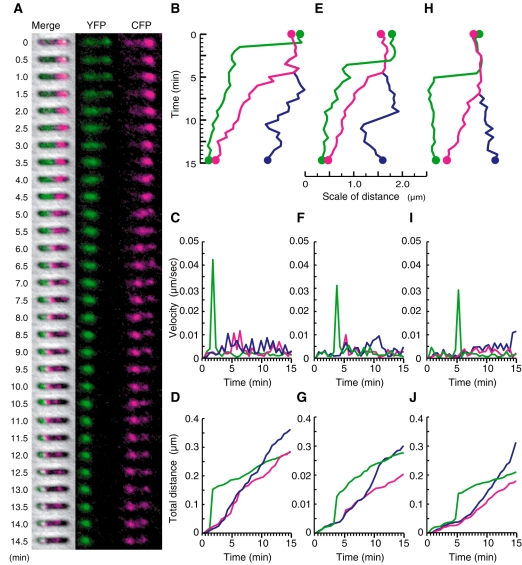
Analysis of movement of SopA and plasmid DNA during separation of the plasmid A. Time lapse images of SopA-YFP and LacI-CFP (plasmid DNA). TH456 cells carrying pNIG113 (*sopA-yfp, sopB*^+^*C*^+^, *lacO*) were exponentially grown at 30°C in M9 succinate medium with supplements. Images were captured for 14.5 min with intervals of 30 s, and each frame is shown. Merged images showing phase contrast images, fluorescence images of SopA-YFP (YFP), and fluorescence images of LacI-CFP (CFP) are shown (Merge). B, E and H. Kymographs showing the movement of SopA-YFP (green), and LacI-CFP (magenta and blue). Scale bar indicates 2.5 μm, as the distance of migration. C, F and I. Velocity of movement of SopA-YFP (green) and LacI-CFP (magenta and blue). D, G and J. Total travel distance of SopA-YFP (green) and LacI-CFP (magenta and blue). Analyses of movements in (A) are shown in (B–D), and others are shown in (E–G) and (H–J).

We monitored plasmid segregation by monitoring the separation of a single fluorescent focus of LacI-CFP in a living cell (Fig. 5A). Figure 5B and Fig. S4B show kymographs of the movements of both SopA-YFP and LacI-CFP foci during plasmid segregation. After transfer of the SopA-YFP focus to the distal end, the single LacI-CFP focus remained at the cell pole for a few minutes and did not immediately follow the SopA-YFP focus. This is in contrast to the oscillating phase in which LacI-CFP focus started to move soon after transfer of the SopA-YFP focus to the other end (Fig. 4B). Suddenly, the single fluorescence focus of LacI-CFP was split into two foci. Although one of the foci continued to move, it stayed around the position at which the focus became separated. Whereas the other started to travel to the opposite pole, at which the SopA-YFP focus was located. Interestingly, both the separated foci moved with similar velocity after separation (Fig. 5C), and the cumulative travel distances of the LacI-CFP foci increased roughly in proportion to the duration of the movement (Fig. 5D). Furthermore, the cumulative travel distance of the ‘stationary’ focus appeared to be greater than that of the focus that travelled to the other end in the examples we observed. These results indicate that the ‘stationary’ plasmid DNA copy was not constantly anchored near the cell quarter, and that the position of the plasmid was fluctuated around this area. In contrast, the ‘travelling’ plasmid DNA copy moved to the opposite pole directly without varying from its path. An additional two kymographs of plasmid separation showed similar movements for LacI-CFP and SopA-YFP (Fig. 5E–J).

### Filamentous structure of SopA

**Fig. 6:**
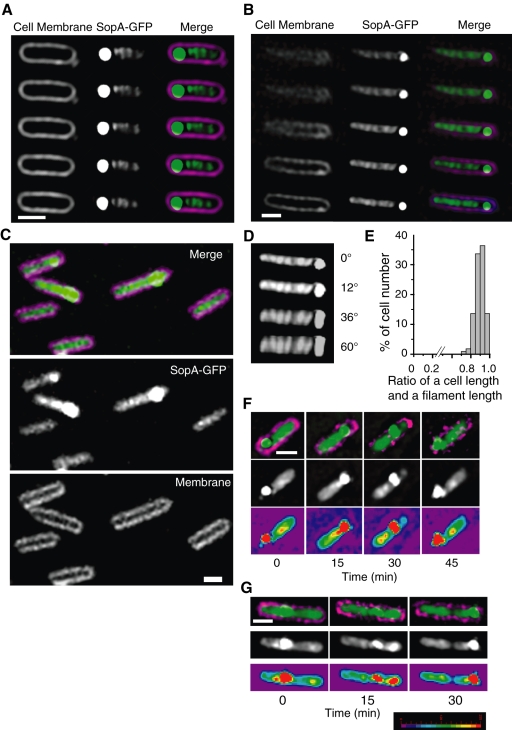
Characterization of the SopA filaments in cells. TH456 cells carrying pNIG105 (*sopA-gfp*, *sopB*^+^*C*^+^) were exponentially grown at 30°C in M9 succinate medium with supplements. The cell membrane was stained with FM4-64. A and B. Sectional images of SopA-GFP in a cell fixed with 80% methanol (A) and a living cell (B). The images were captured along the *z*-axis at 0.2-μm intervals, and treated with a deconvolution algorithm. Merged images showing SopA-GFP (green) and the cell membrane (magenta) are shown (Merge). Scale bars indicate 1 μm. C. An optical section image of SopA-GFP in a living cell obtained by confocal microscopy at 0.1-μm intervals along the *z*-axis of the microscope. (Top) Merged images showing SopA-GFP (green) and the cell membrane (magenta). (Middle) SopA-GFP. (Bottom) Cell Membrane. Scale bar indicates 1 μm. D. A three-dimensional image of SopA-GFP was reconstructed based on the images shown in panel B and visualized at the angle of degree as indicated. E. Ratio of the length of the SopA-GFP filaments to cell length. Cells (*n* = 110) that contained the SopA-GFP filament without a focus were measured. F and G. Time-lapse observation of the filamentous SopA-GFP structure. Deconvolved sectional images of living cells are shown as projection images. The sectional images were taken every 15 min. A unit cell (F) and a dividing cell (G). Merged images of SopA-GFP (green) and the cell membrane (magenta) (top), SopA-GFP alone (middle), and distribution of the relative signal intensity of SopA-GFP (bottom) are shown. The relative signal intensity between 0 and 255 in a cell is shown as a gradation of colour from magenta (weakest) to red (strongest) according to the colour gradient bar shown at bottom right. Scale bar indicates 1 μm.

A single dense fluorescent SopA-YFP focus was visualized in cells by a conventional fluorescent microscopy. Higher-sensitivity microscopic imaging at combined with deconvolution algorithms revealed a SopA-YFP filamentous shaped structure along the long axis of both fixed and living cells (Fig. 6A and B). We could also detect the filamentous structure of SopA-YFP by confocal microscopy, indicating that the filamentous shape of deconvolved images we had shown here were not artefacts produced by deconvolution algorithms (Fig. 6C). In some cells, the SopA-YFP focus was very bright at the pole and the filamentous structure appeared throughout the cytoplasm (Fig. 6A), while in others, only the filamentous structure extended to the tip of the cell pole (Fig. 6C). Three-dimensional analysis confirmed that the continuous filamentous structure in cells (Fig. 6D). We analysed a number of cells with the filamentous SopA-GFP structure (Fig. 3A). Although we failed to detect filamentous structure in 20.4% of the cells, almost all the cells harbouring SopA-GFP foci also had the filamentous SopA-GFP structure. Interestingly, a significant number of cells (24.3%) had a filamentous SopA-GFP structure without a bright focus (Fig. 3A; top row), suggesting SopA-GFP was in the process of being transferred from one pole to the opposite pole, or the cell just divided without inheriting the strong focus and did not have enough time to develop one. The filamentous structure in cells without the SopA focus reached from pole to pole in almost all cells. In addition, various mutations of the *sopABC* genes inhibited the assembly of SopA on the nucleoid tips (Fig. 3C–G), and also prevented formation of the filamentous structure of SopA-YFP (data not shown). It appears that formation of the filamentous structure of SopA is associated with the process of the active partitioning of the F plasmid.

### The length of filamentous structure remains constant during oscillation of SopA

If elongation (or contraction) of the filamentous SopA structure provides the motive force for plasmid migration, the length of the SopA filament should change as the plasmid migrates. To address this question, we examined the relative relationship between cell length and filament length among cells harbouring a filamentous SopA structure without SopA foci (Fig. 3A; top row). Figure 6E shows that the ratio of cell length to filamentous length was more than 0.8 in almost all cells. This result indicates that the SopA-GFP protein assembles in a filamentous structure that span the entire length of the cell without becoming shorter as the plasmid migrates. However, the above result cannot eliminate the possibility that the filamentous filaments temporarily contracted for a short period during oscillation of the SopA foci. Therefore, we analysed the dynamics of the filamentous structure in living cells in which oscillation of the SopA focus was occurring (Fig. 6F). At each time point, the filamentous structure extended from cell pole to cell pole. Although the structure of the SopA filament as a whole remained constant, the intensity distribution of GFP fluorescence did change slightly (Fig. 6F). This result suggests that the SopA proteins that comprise the filaments are not static and that redistribution of the SopA protein possibly occurs within the filament while maintaining the overall structure. Furthermore, in dividing cells, the filamentous structure was also divided at midcell (Fig. 6G). These results indicate that the filamentous structure spans the cell length and gradually elongates and divides following the cell growth and division. This observation contradicts the model in which the filament acts as an elongating or contractile apparatus that pushes or pulls the plasmid DNA.

### Formation of the SopA filament in anucleated cells

Because we found that the SopA foci tend to be localized on nucleoid tips, it seemed plausible that the SopA protein bound to host chromosome DNA at nucleoid tips as a nucleation site for focus formation, because the SopA protein is able to bind to DNA and function as the auto-repressor of its promoter. This hypothesis implies that a nucleoid is an essential factor for positioning and oscillation of the SopA foci. If so, regular positioning and oscillation of the SopA foci would be disturbed in anucleated cells (DNA-less cells). We therefore investigated how the SopA-YFP foci behaved in anucleated cells. We previously reported that mutants with a large chromosomal inversion between the Ori domain and the Ter domains produces anucleated cells at a high frequency (Niki *et al*., 2000). However, a suppressor mutation, a secondary inversion between the IS elements on the chromosome, easily occurs in the mutants with the large inversion during cultivation (Cornet *et al*., 1996), and the ratio of anucleated cells is decreased overall. Thus, we constructed a strain in which mutants with the large chromosomal inversion could be selected as antibiotics resistant cells, which produced anucleated cells at a higher frequency (19.6%: 227/1160 anucleated cells).

**Fig. 7:**
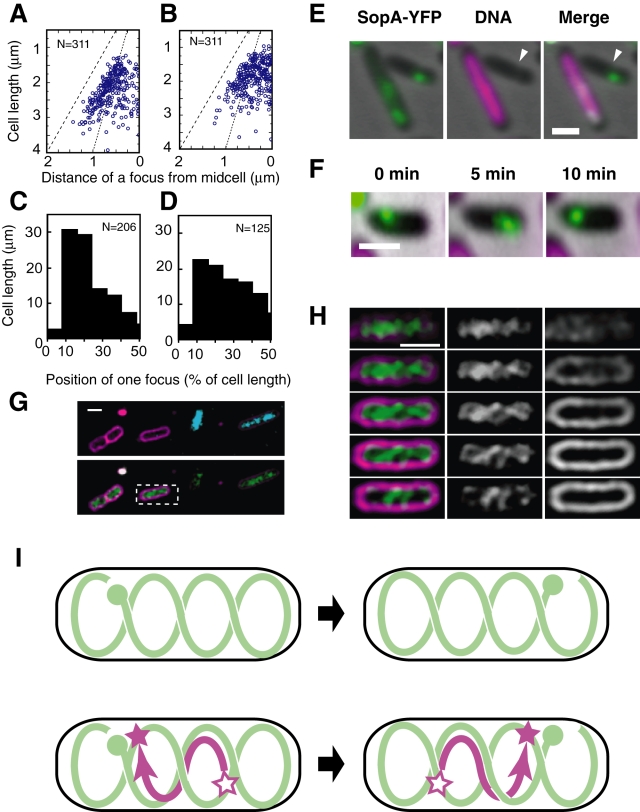
Subcellular localization of SopA-YFP in anucleated cells. Cells were exponentially grown at 30°C in M9 medium including succinate with supplements, and treated with 1 μg ml^−1^ Hoechst 33342 to stain DNA for 1 h A and B. Position of the SopA-YFP focus in the parent strain, TH768 (YK1238/pNIG111) (A), or in anucleated cells, TH790 (B). Position in cells with a single focus is plotted versus cell length. The dotted lines indicate the middle of the cells and dashed lines indicate the position of a pole. C and D. Histograms showing the distribution frequency of the foci in cells longer than 2 μm with a single focus. Normal cells (C) and anucleated cells (D) are shown. E. Localization of SopA-YFP in cells with or without nucleoids (anucleate cell). Phase contrast image merged with SopA-YFP fluorescence images (green; left), chromosomal DNA image (magenta; middle), and with both SopA-YFP fluorescence (green) and chromosomal DNA image (right) are shown. The arrowhead indicates an anucleated cell. F. Time lapse images of SopA-YFP fluorescence in an anucleated cell. Images were captured for 10 min at intervals of 5 min. Phase contrast images merged with both SopA-YFP fluorescence (green) and chromosomal DNA images (right) are shown. G. Projection images of sectional images of SopA-YFP fluorescence in an anucleated cell. Sectional images were captured along the *z*-axis with 0.2-μm intervals and treated with a deconvolution algorithm. The deconvolved image of the cell membrane (magenta) was merged with that for chromosomal DNA (blue; upper), or for SopA-YFP fluorescence (green; lower). H. A series of sectional images of an anucleated cell with the SopA-YFP helical structure (surrounded by a white dashed rectangle in the lower panel of G). Merged images of the cell membrane (magenta) and SopA-YFP fluorescence (green: left), SopA-YFP fluorescence alone (middle) and the cell membrane alone (right). Scale bars indicate 1 μm. I. A model of the helical structure of SopA (green) and movement of F plasmid (magenta star) in a cell. SopA assembled near the cell pole is shown as circle. The polarity of the filament is changed during oscillation of SopA. The polarity change switches the direction of the movement of F plasmid (magenta arrow).

We next examined whether SopA was localized at the cell poles. We found that about 17% of anucleated cells had the SopA-YFP foci at cell poles (39 cells/227 anucelated cells). Of the anucleated cell producing cells, about 23% of cells with nucleoids also had the SopA-YFP foci (216 cells/933 cells), and about 17% of the parental cells had the SopA-YFP foci (218 cells/1294 cells); these ratios were not significantly different. We next analysed the subcellular location of the SopA-YFP foci in the parent cells and anucleated producing cells (Fig. 7A, B and E), and found position of the SopA-YFP single foci seemed to be distributed over anucleated cells, as seen in Fig. 7B. When the distribution of the SopA-YFP foci was analysed carefully, the SopA-YFP foci tended to be more often positioned near the cell poles. This was clearer in longer cells (> 2 μm). The SopA-YFP foci could be localized to a position at 10–20% along the cell length in anucleated cells, as in wild-type cell (Fig. 7C and D). Moreover, the SopA foci still oscillated in anucleated cells. Each of the SopA foci in living cells with or without nucleoid changed the position, suggesting oscillation of the SopA foci is independent of the nucleoids (Fig. 7F). In addition to the location of the SopA-YFP foci, we investigated the formation of the filamentous structure in anucleated cells and detected the filamentous structure in these cells (Fig. 7G and H). These results clearly indicate that the nucleoid is not essential for the assembly, positioning, or formation of filaments of SopA.

## Discussion

To avoid interference with the physiological state of segregating plasmids, SopA-GFP or SopA-YFP were under control of the native *sop* promoter on the mini-F plasmid (Fig. 1) and then expressed at as low level as possible (Fig. 2). To analyse the movements of plasmid DNA and the SopA protein in a single cell for as long as possible, we developed a system to detect faint fluorescence in this experiments. Thus, we succeeded in taking time-lapse photographs for at least 15 min. Oscillation of SopA was observed in almost all cells with the SopA foci. Oscillating periodically, as has also been observed for the Min proteins (Raskin and de Boer, 1999), the Soj proteins (Marston and Errington, 1999; Quisel *et al*., 1999), and SopA (Lim *et al*., 2005). Oscillation of SopA was similar to those of the Min proteins and the Soj proteins, except for the interval of oscillation. The interval of oscillation of Min proteins is less than 1 min (Raskin and de Boer, 1999). In contrast, the intervals of oscillation of the SopA foci varied from a few minutes to more than 10 min in each cell. The intervals were similar to that of the Soj proteins. Longer interval of the SopA oscillation (about 20 min) was monitored (Lim *et al*., 2005). Oscillation of SopA is slower than that of the Min proteins, suggesting regulatory mechanism of oscillation is different from each other. Timing of the SopA oscillation might be regulated by the SopB–*sopC* complex on the plasmid as shown in Fig. 4A and B. Therefore, this indicates that velocity of the plasmid including the SopB–*sopC* complex may be one of crucial factors to determine a period of the SopA oscillation.

The oscillation of SopA is compatible with the reaction-diffusion partitioning model that proposed by Adachi *et al*. (2006). However, we are unable to straightly apply this model to some of our results. First, the model could not explain the dynamic change of direction of the F plasmids movement by the SopA (Fig. 4A and B). Second, when the replicated plasmids split near the one pole, one of the daughter plasmids migrated towards the opposite pole. In addition to the SopA oscillation, the SopB–*sopC* complex on F plasmid could be oscillated in a cell (Fig. 4A and B). Thus, two oscillating components of the *sopABC* partitioning system are open to further discussion on theoretical models that is based on reaction-diffusion equations.

In this study we attempted to understand how the putative motor protein SopA contributes to migration of the actively partitioning plasmids. Bidirectional extension of the ParM filament with plasmids at both ends during plasmid segregation (Moller-Jensen *et al*., 2003; Garner *et al*., 2004) has been observed. Although Adachi *et al*. (2006) also observed the filamentous structure of SopA by indirect immunofluorescence microscopy in fixed cells, dynamics of the SopA filaments had not been investigated. SopA has been shown to form filaments *in vitro* in an ATP and SopB-dependent manner (Lim *et al*., 2005; Bouet *et al*., 2007). These filaments polymerize or extend *in vitro*, and the average rate of *in vitro* polymerization is similar to the rate of F plasmid separation (Lim *et al*., 2005). This observation suggests that SopA polymerization is coupled to partition. However, the SopA polymerization *in vivo* has not been demonstrated until now. We found that SopA formed a filamentous structure in a living cell (Fig. 6B, C, F and G). Our results of time-lapse observation found no clear evidence for extension and retraction of the filament in cells with actively partitioning plasmids (Fig. 6F and G). Instead, we provide evidence showing that the relative length of the filament remains constant (Fig. 6E). These results are insufficient to completely eliminate the possibility that filaments temporarily contracted for a short period during oscillation of the SopA foci. Alternatively, if filament elongation and retraction rates are in equilibrium (i.e. tread-milling by assembly and disassembly at opposite ends), then a filament will remain a constant length.

The plasmid may migrate on the SopA filament in a cell. We consider that the SopA filament guides the plasmid to the cell quarters as if the SopA filament were a railway track (Fig. 7I). In addition, the SopA filament may be given polarity, and then plasmid migrates towards the one direction. SopA dynamics are likely important for plasmid partitioning, as a SopA static mutant is defective for partition (Lim *et al*., 2005). Thus, it is not merely the ability of SopA to form filaments that is crucial for partition, they must be dynamic, and be able to reversibly polymerize and depolymerize. The dynamics of SopA probably contributes to the polarity change of the SopA filaments in a cell. Perhaps the bright SopA foci are considered as a nucleation core that assembles near one end of the filament (Fig. 7I). The oscillation of SopA foci causes a change in the polarity of the filament, so that the direction of plasmid movement is switched from one cell pole to the other. Thus, polarity of the SopA filamentous structure might be important for determining the direction of the plasmid movement.

It is observed that SopA forms radial aster *in vitro* (Lim *et al*., 2005). This also suggests that multiple fibres of SopA can form in cell. Therefore, it seemed that these filaments that extend to radial pattern from SopA foci, a nucleation core, looked amorphous structure in cells (Fig. 6).

In general, a motor protein converts chemical energy into physical energy, and contributes to generating driving force or directional movement against thermal fluctuation. In this context, the SopA ATPase could generate driving force for plasmid movement. The biochemical property of SopA suggests that when the plasmid with the SopB–*sopC* complex approaches the assembled SopA near the cell pole, the SopB–*sopC* complex could enhance the ATPase activity of SopA. The stimulation of ATPase activity could further enhance the disassembly of SopA, and then SopA could reassembles at the opposite cell pole. Although assembly and disassembly of SopA foci represent abrupt changes, the migrating plasmid has an almost constant velocity.

When the SopB–*sopC* complex stimulates the SopA ATPase activity, SopA can inversely disrupt the SopB–*sopC* complex by interacting with SopB (Lemonnier *et al*., 2000). The actively partitioning plasmid harbouring the SopB–*sopC* complex follows the oscillating SopA, and reaches to the assembled SopA. This might correspond to colocalization of the SopB and *sopC* complex with SopA (Lim *et al*., 2005). Then deterioration of the stable complex of the SopB and *sopC* complex may cause the actively partitioning plasmid to pause and change direction. In the case of paired plasmids, the plasmids could separate from each other by disruption of the SopB–*sopC* complex. SopB cooperatively binds to the *sopC* DNA (Mori *et al*., 1989), thus one of the plasmids may easily recover the SopB–*sopC* complex and continue to follow the SopA, and the other may not. Thus, the interaction between SopA and the SopB–*sopC* complex is responsible for directional movement and segregation of actively partitioning plasmids. This indicates that the cellular amount of SopA and SopB is crucial for the dynamic SopABC partitioning system. In fact, increases in either SopA or SopB cause a defect in proper partitioning of the F plasmid (Ogura and Hiraga, 1983; Kusukawa *et al*., 1987).

Previous works suggest that the F plasmid is tightly attached at the cell quarters (Gordon *et al*., 1997; Niki and Hiraga, 1997; Shih *et al*., 2006; Ebersbach and Gerdes, 2005). This suggests that the plasmids bind to a host factor at the cell quarters in host cells. However, the identities of the host factors for the plasmid localization have remained unknown until now. In the present study, the plasmid DNA moved backwards and forwards along long axis of the cell length (Fig. 4A and B), but tended to be localized at the middle of cells (Fig. 4E). It is possible that the wandering of the focus represents not net movement of the whole plasmid but reorientation or successive relaxation and compression of a fixed plasmid. In any case, the SopA filament could help to localize the plasmid in a specific subcellular region.

In fact, our results also indicate that SopA tends to assemble at the specific subcellular sites. SopA oscillation in anucleated cells occurred between positions at which nucleoid tips may be located if there is nucleoid in the cell (Fig. 7B and D). We cannot completely exclude the possibility that one or more unknown host factors are involved in positioning of SopA foci. It is likely that SopA dynamics is critical for its localization and positioning of the SopB–*sopC* complex at the cell quarters.

The P1 prophage has a ParABS partitioning system, and the ParA ATPase and the ParB DNA binding protein belong to the same protein families as SopA and SopB, respectively (Yamaichi and Niki, 2000). Previous works based on biochemical and genetic studies have indicated that the ParABS partitioning system and the SopABC partitioning system have common molecular mechanisms (Hiraga, 2000; Gordon and Wright, 2000; Draper and Gober, 2002). However, movement of the plasmid with the SopABC partitioning system is different from that with the ParABS partitioning system (Li and Austin, 2002; Li *et al*., 2004). Plasmid segregation by ParA of the pB171 plasmid occurs as well as P1 plasmid segregation. ParA of pB171 is a member of the Walker type ATPase family, and can oscillate from pole to pole. In addition, the focus of ParA is localized at the nucleoid border (Ebersbach and Gerdes, 2004). Thus, ParA of pB171 has similar properties to SopA. The growth conditions of the host cells may also affect movement of the plasmid, and the *parS*/GFP-ParB tagging method used to analyse plasmid localization can also alter some aspects of plasmid dynamics (Gordon *et al*., 2004). It is possible that, GFP-ParB may have a tendency to localize at midcell, because the SopB, an orthologue of ParB of P1, has the intrinsic property of specific localization at midcell (Kim and Wang, 1998; Yamaichi and Niki, 2004). However, the partitioning dynamics of the P1 and F plasmids are very different. ParA-CFP of the P1 plasmid does not oscillate from pole to pole, and ParA-CFP is distributed on nucleoids with a single faint focus (T. Hatano *et al.*, unpubl. data). This suggests that there are mechanical differences between the partitioning systems.

In the present study, almost all SopA-YFP proteins were assembled as a single focus located on one of the separated nucleoids in a dividing cell. Thus, the subcellular distribution of SopA caused asymmetrical inheritance of SopA-YFP during cell division as occasionally almost all SopA was allocated to one of the daughter cells (Fig. S3B). This explains why some fractions of cells do not have significant fluorescence regardless of the high retention rate of the plasmid in cells (Fig. 3A). This result suggests that asymmetric inheritance of the oscillating proteins including MinD and Soj could be seen in a dividing cell. Hence, oscillation of SopA and its homologues could be considered as one of the fundamental mechanisms by which asymmetrical distribution of proteins occurs in prokaryotes or eukaryotes.

## Experimental procedures

### Bacterial strains and plasmids

The bacterial strains and plasmids used in this study are listed in Supplemental Table S1. The *sopA* fusion gene under control of the sop operator was constructed according to the procedures of Datsenko and Wanner (2000) and of Yu *et al*. (2000) (Fig. 1 and Fig. S1), as described in detail in the Supplemental Text.

### Growth conditions and media

Cells were grown in L broth or M9 medium supplemented with sodium succinate (0.25%), thiamine (1 μg ml^−1^), leucine (50 μg ml^−1^), arginine (50 μg ml^−1^), proline (50 μg ml^−1^) histidine (50 μg ml^−1^) and threonine (50 μg ml^−1^). The following antibiotics were added to the medium when necessary: ampicillin (20 μg ml^−1^ or 100 μg ml^−1^), chloramphenicol (15 μg ml^−1^) and kanamycin (15 μg ml^−1^). The generation time (doubling time) was determined on the basis of the turbidity of cultures.

### Fluorescence microscopy and optical sectioning

Cells from overnight cultures grown at 30°C or 37°C in the M9 medium were diluted and grown at 30°C or 37°C to the early exponential phase. Expression of the LacI-CFP fusion protein was induced by addition of 0.2% arabinose to the media for 3 h. Optical sectioning experiments were performed using an Olympus IX70 microscope with a PlanApo X100 1.40 oil immersion objective lens and the Delta Vision system (Applied Precision). Microscopic imaging was carried out as described in detail in the Supplemental Text.

## Supplementary material

The following supplementary material is available for this article:

Construction of Bacteria strains and plasmids, methods used in this study are described in detail.

Fig. S1 Construction of the *sopA-gfp* fusion gene on a mini-F plasmid. H1 and H2 indicate the homology extensions or regions. FRT indicates an FLP recognition target. SD indicates a putative Shine-Dalgarno sequence. TAA indicates the stop codon of the *sopA* gene.

Fig. S2 Expression of SopA-YFP/GFP fusion proteins. TH456 cells harbouring the plasmids were exponentially grown and lysed as described in supplemental text. Indicated volume (μl) of the lysed sample were subjected to SDSpolyacrylamide gels electrophoresis, followed by Western blot analysis using rabbit anti-SopA antibody for detection of SopA protein (A) and Living Colors™ A.v. peptide antibody for detection of GFP or YFP fusion protein (C). B. Chemical luminescence signals of each band of proteins, SopA (43.7 kDa), SopA-YFP (70.7 kDa) and SopA-GFP (70.7 kDa) in (A) were quantified and plotted in a graph.

Fig. S3 Subcellular localization of SopA-GFP and SopAYFP. A. Localization of SopA-GFP in a cell fixed in 80% methanol. TH456 cells carrying pNIG105 (*sopA-gfp*, *sopB*^+^*C*^+^) were exponentially grown at 30°C in M9 succinate medium with supplements, and fixed in 80% methanol. All images are combined with a phase contrast image to illustrate cell morphology. Fluorescent foci of SopA-GFP and nucleoids are shown in green and magenta respectively. Merged images with SopA-GFP and nucleoids are shown (Merge). Scale bar indicates 1 mm. B. Distribution of a single focus of SopA-GFP in a dividing cell. TH456 cells carrying pNIG105 (*sopA-gfp*, *sopB*^+^*C*^+^) were grown to log phase at 30°C in M9 succinate medium with supplements. Living cells with SopA-GFP were observed by using a deconvolution system. The cell membrane was stained with FM4-64 to detect the septum in dividing cells. Scale bar indicates 1 μm. C. Histogram showing the distribution frequency of the foci in cells with a single focus of SopA-YFP expressed from pNIG113 (*sopA-yfp*, *sopB*^+^*C*^+^, *lacO*).

Fig. S4 The traces of the fluorescent foci of SopA-YFP and LacI-CFP. A and B. The traces of centre of the fluorescent foci of SopA-YFP and LacI-CFP in a series of time-lapse pictures in Figs. 4A and 5A respectively.

Table S1 Bacterial strains and plasmids used in this study.

This material is available as part of the online article from: http://www.blackwell-synergy.com/doi/abs/10.1111/j.1365-2958.2007.05728.x (This link will take you to the article abstract).
