# Supplementary material for: Oscillating focus of SopA associated with filamentous structure guides partitioning of F plasmid
Source: Mol Microbiol. 2007 Jun 1;64(5):1198–213. doi: 10.1111/j.1365-2958.2007.05728.x (PMC1974783; doi:10.1111/j.1365-2958.2007.05728.x)
Supplement: Fig. S1 — Construction of the sopA-gfp fusion gene on a mini-F plasmid. H1 and H2 indicate the homology extensions or regions. FRT indicates an FLP recognition target. SD indicates a putative Shine-Dalgarno sequence. TAA indicates the stop codon of the sopA gene. [file mmi0064-1198-S1.pdf]

Supplemental Text

Construction of plasmids

The plasmids used in this study were constructed as follows. To express the *sopA*-*gfpUV4* fusion gene under the control of the sop operator, the stop codon of *sopA* was replaced in frame with the *gfpUV4* gene in accordance with the procedures of Datsenko and Wanner (2000) and Yu et al. (2000) (Fig. S1). First, an FRT (FLP Recognition Target)-flanked kanamycin resistance cassette was amplified by PCR and cloned into the EcoRI site of pGFP^{uv4} (Ito et al. 1999). For the PCR, pKD4 template DNA (Datsenko and Wanner. 2000) and a set of primers as follows were used: TPR-040, 5'-ccggaattctgtgtaggctggagctgcttc-3' and TPR-041, 5'-ccggaattccatatgaatatcctcctta-3'. The cloned plasmid was named pTH5. The DNA fragment containing both *gfpUV4* and the FRT cassette was amplified using pTH5 as a template with a forward primer containing the last 40 bp of *sopA* except for the stop codon and the first 20 bp of *gfpUV4* (TPR-32, 5'-aattttcgatcgtctgattaaaccacgctgggagattagaatgagtaaaggagaagaact-3') and a reverse primer containing 40 bp of *sopB*, the putative SD region upstream of *sopB* and priming site 1 in pKD4 (Datsenko and Wanner. 2000) (TPR33, 5'-tattgagcgtatgttttggaataacaggcgcacgcttcattttattatctcccgtgtaggctggagctgcttc-3'). The PCR product was electroporated into TH48 cells (DY330 harboring pXX325) and screened in accordance with the method of Yu et al. (2000). Then, the FRT-flanked kanamycin resistance cassette was eliminated using an FLP expression plasmid, pCP20, resulting in pNIG105.

In the same way, the plasmid expressing the SopA-YFP fusion protein was generated. First, the template plasmid, pTH64, was constructed by inserting the FRT-flanked kanamycin resistance cassette into the *Spe*I site of pEYFP (BD Biosciences) using a set of primers as follows: TPR-9, 5'-ggactagtgtgtaggctggagctgcttc-3' and TPR-10, 5'-ccactagtcatatgaatatcctcctta-3'. Then, the DNA fragment containing the *eyfp* gene-FRT-flanked kanamycin resistance cassette was PCR amplified using pTH64 as the template, with a forward primer containing the first 20 bp of the *gfp* gene except the first Met codon (TPR42, 5'-aattttcgatcgtctgattaaaccacgctgggagattagagtgagcaagggcgaggagct-3'), and TPR33 as the reverse primer. The DNA fragment was introduced into pXX325, and the kanamycin-resistant cassette was eliminated as described above, resulting in pNIG111.

A plasmid containing the *lac* operator (*lacO*) cassette was constructed as follows. A DNA fragment containing the *lacO* cassette was isolated from pSTV2-8.32 (gift from A. Wright) digested with *Sal*I and *Xho*I, and cloned into pXX325 digested with *Sal*I, resulting in pNIG100. To allow simultaneous observation of both the SopA-YFP fusion protein and *lacO*/LacI-CFP in a living cell, a plasmid (pNIG113) harboring both the *sopA-gfp* fusion gene and *lacO* cassette was constructed as described above using pNIG100. Deletion mutants of the *sop* loci were constructed in accordance with the methods of Yu et al. (2000) and Datsenko and Wanner (2000). Each deletion was confirmed by PCR analysis. The deleted regions are detailed in Supplemental Table I. Genetic coordinates were determined by Mori et al. (1986).

Construction of bacterial strains

The strains used in this study are listed in Supplemental Table I. To express the *lacI-ecfp* fusion gene (Lau et al. 2003) at an appropriate expression level in cells, the fusion gene under control of the arabinose promoter was placed on the chromosome. The *tetR-ecfp* gene and ampicillin resistance gene in pLAU53 were deleted and replaced with the FRT-flanked kanamycin resistance cassette using pKD4 as a template with primers as follows: TPR043, 5'-cctgataaatgcttcaataatattgaaaaaggaagagtcatatgaatatcctcctta-3' and TPR046, 5'-atgatgtctagattagataaaagtaaagtgattaaccatatgaatatcctcctta-3'. The resulting plasmid was named pTH118. Next, a DNA fragment containing *araC*, *lacI-ecfp* and the FRT cassette was amplified by PCR using pTH118 as a template with primers as follows: TPR047, 5'-gtcgttggatcagatccgccaggcgatggaagagtaggttttatgacaacttgacggcta-3' and TPR48, 5'-tcggcacacagtgccggatgcaccgcgcgacgcatccgactatatgagtaaacttggtct-3'. The PCR product was replaced with non-cording nucleotides 353820-353822 on the chromosome in the AB1157 strain (genetic coordinates are from the Microbial Genome Workbench; http://www.bioscinet.org/). The resultant strain, TH130, expressed the LacI-CFP proteins upon addition of 0.2% arabinose. The FRT cassette in TH130 was eliminated as described above, resulting in TH150. To improve expression of LacI-CFP, the *lacIZYA* operon region on the chromosome was deleted and replaced with the FRT-flanked chloramphenicol resistance cassette amplified using pKD3 as a template (Datsenko and Wanner. 2000) with primers TPR051 (5'-ggcgcaaaacctttcgcggtatggcatgatagcgcccggagtgtaggctggagctgcttc-3') and TPR052 (5'-aactgacgattcaactttataatctttgaaataatagtgccatatgaatatcctccttag-3') in accordance with the method described by Datsenko and Wanner (2000). The resultant strain was named TH456.

Fluorescence microscopy

Cells from overnight cultures grown at 30°C or 37°C in M9 medium were diluted and exponentially grown at 30°C or 37°C. Expression of the LacI-CFP fusion protein was induced by addition of 0.2% arabinose to the media for 3 hours. Living cells were then mounted in M9 medium on a 0.1% poly-L-lysine-coated coverslip. To allow observation of the cell membrane, cells were incubated for 15 min in culture with 0.4 M N-(3-triethylammoniumproryl)-4-(6-(4-(diethylamino)phenyl)-hexatrienyl) pyridinium dibromide (FM4-64; Molecular Probes). To allow localization of nucleoids, cells were fixed with 80% methanol at 4°C for 10 min and stained for 30 min with 1 μg/ml 4', 6-diamidino-2phenylindle (DAPI; Dotite), then 1 mg/ml p-phenylenediamine dihydrochloride (Sigma).

Optical sectioning

Living or fixed cells expressing the SopA-GFP fusion protein and its derivatives were fixed on a glass-bottomed dish (Matsunami Glass Inc., Japan) that was coated with 0.1% concanavalin A (ConA; Vector Laboratories), and the cells were embedded in 0.1% low melting point agarose containing M9 medium with supplements (as detailed in the Experimental Procedures section). Optical sectioning experiments were performed using an Olympus IX70 microscope with a PlanApo X100 1.40 oil immersion objective lens and the Delta Vision system (Applied Precision). Sets of optical section images were obtained at 0.2-μm intervals for living cells and at 0.05-μm intervals for fixed cells, along the z-axis of the microscope. Each stack of 10-20 optically sectioned images was deconvolved through 20 iterations using the SoftWorx version 2.50 program (Applied Precision). Although

SopA-GFP was slightly affected to stability of the plasmid partitioning in Fig 1B, localization of SopA-GFP was very similar to that of SopA-YFP. As modified version of GFP or GFPuv4 can emit much brighter fluorescence than YFP, we could obtain clearer fluorescence images of SopA-GFP by the Optical sectioning experiments. The stacks of deconvolved images were reconstituted to three-dimensional images using the "volume view" function of the program. Optical sectioning experiments by confocal microscopy were performed using a Zeiss LSM 510 microscope with a Zeiss PlanApocromato X100 1.40 oil immersion objective lens (Fig. 6C). Sets of optical section images were obtained at 0.1-μm intervals for living cells along the z-axis of the microscope.

Time-lapse imaging

Cell were exponentially grown at 30°C in the M9 medium supplemented with sodium succinate (0.25%), thiamine (1 μg/ml), leucine (50 μg/ml), arginine (50 μg/ml), proline (50 μg/ml) histidine (50 μg/ml). and threonine (50 μg/ml). The cells were transferred to a glass-bottomed dish coated with concanavalin A (0.1%). Living cells incubated at 30°C were observed under an inverted microscope (Axiovert 200M; Zeiss, Germany) fully controlled by a PC running Axiovision software (Zeiss, Germany). Images were captured using an Axiocam charge-coupled device (CCD) camera system. An image intensifier unit (C8600-03; Hamamatsu, Japan) was used to reduce the intensity of the excitation light. The images were improved using Adobe Photoshop ver. 7 software. Central points of each focus were traced to determine velocity and travel distance by AQACOSMOS software (Hamamatsu, Japan).

Plasmid stability assays

Cells harboring plasmids were grown in L broth containing ampicillin (20 μg/ml) overnight at 37°C and diluted with fresh L broth containing ampicillin and grown to early log phase for 1-2 hours at 37°C. The culture was diluted with fresh L broth without ampicillin and incubated at 37°C, keeping cells exponentially growing by dilution with fresh L broth. Aliquots of the culture at each time point were plated on L agar plates and incubated at 37°C. Colonies were streaked on L agar plates containing ampicillin (20 μg/ml) to test for the presence of the plasmid.

Western blotting

Cells were exponentially grown at 30°C in L medium. Appropriate amount of cells were diluted in Laemmli sample buffer and boiled for 3min. The samples were subjected to 10% SDS-polyacrylamide gels electrophoresis. Separated proteins were transferred to a polyvinylidene difluoride membrane (MILLIPORE). Blotted membranes were incubated for overnight at 4 ˚C with rabbit anti-SopA antibody or Living Colors ™ A.v. peptide antibody (Takara) for GFP or YFP protein, and followed by washing with 10 mM PBS/0.1% Triton-X-100. Subsequently, incubation with horseradish peroxidase-coupled goat anti-rabbit antibody was performed for 1 h at room temperature, and followed by washing with 10 mM PBS/0.1% Triton-X-100. Blots were finally analyzed using the ECL Plus Detection System (Amersham Biosciences, San Francisco, CA, USA). The fluorescent signals of each band were detected by FUJI LAS-1000 Image analyzer (FUJIFILM) and quantified using ImageGauge™ software (FUJIFILM).

References

Datsenko, K.A., and Wanner, B.L. (2000) One-step inactivation of chromosomal genes in Escherichia coli K-12 using PCR products. *Proc Natl Acad Sci U S A* **97**: 6640-6645.

Ito, Y., Suzuki, M., and Husimi, Y. (1999) A novel mutant of green fluorescent protein with enhanced sensitivity for microanalysis at 488 nm excitation. *Biochem Biophys Res Commun* **264**: 556-560.

Lau IF, Filipe SR, Soballe B, Okstad OA, Barre FX Sherratt DJ (2003) Spatial and temporal organization of replicating *Escherichia coli* chromosomes. *Mol Microbiol* **49**: 731-743.

Yu D, Ellis HM, Lee EC, Jenkins NA, Copeland NG Court DL (2000) An efficient recombination system for chromosome engineering in *Escherichia coli. Proc Natl Acad Sci U S A* **97**: 5978-5983.

Fig. S1. Construction of the *sopA-gfp* fusion gene on a mini F plasmid. H1 and H2 indicate the homology extensions or regions. FRT indicates a FLP recognition target. SD indicates a putative Shine-Dalgarno sequence. TAA indicates the stop codon of the *sopA* gene.

Fig. S2. Expression of SopA-YFP/GFP fusion proteins.

TH456 cells harboring the plasmids were exponentially grown and lysed as described in supplemental text. Indicated volume (μl) of the lysed sample were subjected to SDS-polyacrylamide gels electrophoresis, followed by Western blot analysis using rabbit anti SopA antibody for detection of SopA protein (A) and Living Colors ™ A.v. peptide antibody for detection of GFP or YFP fusion protein (C). B. Chemical luminescence signals of each band of proteins, SopA (43.7kDa), SopA-YFP (70.7 kDa) and SopA-GFP (70.7 kDa) in (A) were quantified and plotted in a graph.

Fig. S3. Subcellular localization of SopA-GFP and SopA-YFP.

A. Localization of SopA-GFP in a cell fixed in 80% methanol. TH456 cells carrying pNIG105 (*sopA-gfp*, *sopB*$^+$*C*$^+$) were exponentially grown at 30˚C in M9 succinate medium with

supplements, and fixed in 80% methanol. All images are combined with a phase contrast image to illustrate cell morphology. Fluorescent foci of SopA-GFP and nucleoids are shown in green and magenta, respectively. Merged images with SopA-GFP and nucleoids are shown (Merge). Scale bar indicates 1 μm. B. Distribution of a single focus of SopA-GFP in a dividing cell. TH456 cells carrying pNIG105 (*sopA-gfp*, *sopB*$^{+}$*C*$^{+}$) were grown to log phase at 30˚C in M9 succinate medium with supplements. Living cells with SopA-GFP were observed by using a deconvolution system. The cell membrane was stained with FM4-64 to detect the septum in dividing cells. Scale bar indicates 1 μm. C. Histogram showing the distribution frequency of the foci in cells with a single focus of SopA-YFP expressed from pNIG113 (*sopA-yfp*, *sopB*$^{+}$*C*$^{+}$, *lacO*).

Fig. S4. The traces of the fluorescent foci of SopA-YFP and LacI-CFP.

A and B. The traces of center of the fluorescent foci of SopA-YFP and LacI-CFP in a series of time-lapse pictures in Fig. 4A and 5A, respectively.

Supplemental Table 1. Bacterial strains and plasmids used in this study.

E. coli strain	Relevant genotype (Reference)
AB1157	Wild type (*F- thr-1 leuB6 thi-1 lacY1 galK2 ara-14 xyl-5 mtl-1 proA2 his-4 argE3 rpsL31 tsx-33 supE44 sup-37*) (Bachmann *et al.*, 1972)
MC1061	Wild type (*F- ΔlacX74 rpsL araD139* Δ*(ara leu)7697 galU galK hsdR mcrB thi*)
TH130	the same as AB1157, except Δ(353820-353822)::(*araC lacI-ecfp* FRT-*kan*$^+$-FRT)
TH150	the same as AB1157, except Δ(353820-353822)::(*araC lacI-ecfp*)
TH456	the same as AB1157, except Δ(353820-353822)::(*araC lacI-ecfp*), Δ(*lacIZYA*)::*cat*
DY330	the same as W3110 except Δ*lacU169 gal490 λ cI857* Δ(*cro-bioA*) (Yu *et al.*, 2000)
YK1238	the same as MC1061, except *yieC::*[ka] *narW::*[an] (in this study)
TH768	YK1238/pNIG111 (in this study)
TH790	the same as MC1061, except Δ(*yieC narW*)::(*kan*) /pNIG111 {derived from TH768, recombined by [ka]x[an] -> inverted (84.0-33.0min)} (in this study)

Plasmid	Relevant characteristics
pKD4	*bla*$^+$ FRT-*kan*$^+$-FRT (Datsenko and Wanner, 2000)
pKD3	*bla*$^+$ FRT-*cat*$^+$-FRT (Datsenko and Wanner, 2000)
pCP20	*bla*$^+$ *cat*$^+$ (FLP expression plasmid) (Datsenko and Wanner, 2000)
pLAU53	*bla*$^+$ *araC*$^+$ *lacI-ecfp*$^+$ *tetR-eyfp*$^+$ (Lau *et al*, 2003)
pEYFP	*bla*$^+$ *eyfp*$^+$ (BD Bioscience)
pGFPuv4	*bla*$^+$ *gfpuv4*$^+$ (Ito *et al.*, 1999)
pTH5	pGFPuv4 FRT-*kan*$^+$-FRT (in this study)
pTH64	pEYFP FRT-*kan*$^+$-FRT (in this study)
pTH118	the same as pLAU53, except Δ(*tetR-eyfp bla*)::FRT-*kan*$^+$-FRT (in this study)
pXX325	*bla*$^+$ *sopA*$^+$*sopB*$^+$*sopC*$^+$ (Ogura *et al.*, 1983)
pXX327	*bla*$^+$ Δ*(sopABC)* (Ogura *et al.*, 1983)
pNIG100	pXX325 *lacO*$^+$ (in this study)
pNIG101	pXX327 *lacO*$^+$ (in this study)
pNIG105	pXX325 *sopA*-(J1)*-*gfpuv4*$^+$ (in this study)
pNIG111	pXX325 *sopA*-(J3)*-*eyfp*$^+$ (in this study)
pNIG113	pXX325 *sopA*-(J3)*-*eyfp*$^+$ *lacO*$^+$ (in this study)
pNIG120	pXX325 *sopA*-(J3)*-*eyfp*$^+$, Δ*sopB, sopC*$^+$ (Deletion of 1887-2860 nucleotides

	described in Mori *et al*. 1986) (in this study)
pNIG121	pXX325 *sopA*-(J3)*-*eyfp*$^+$, Δ*sopC, sopB*$^+$ (Deletion of 2861-3450 nucleotides described in Mori *et al*. 1986) (in this study)
pNIG122	pXX325 *sopA*-(J3)*-*eyfp*$^+$, Δ (*sopBC*) (Deletion of 1887-3450 nucleotides described in Mori *et al*, 1986) (in this study)
pNIG138	pXX325 Δ*sopA, sopB*$^+$, *sopC*$^+$, *lacO*$^+$ (Deletion of 721-1510 nucleotides described in Mori *et al*, 1986) (in this study)
pNIG152	pXX325 Δ*sopA, sopB*$^+$, *sopC*$^+$ (Deletion of 721-1510 nucleotides described in Mori *et al*, 1986) (in this study)
pNIG140	pXX325 *sopA*^{K120R}-(J3)*-*eyfp*$^+$ (in this study)
pNIG141	pXX325 *sopA*^{K120Q}-(J3)*-*eyfp*$^+$ (in this study)

*Asterisks indicate type of joint of fusion protein as follows,

J1: C-terminal fusion, the first methionine of fluorescent protein (GFPuv4) was left.

J3: C-terminal fusion, the last amino acid of the fused protein (SopA) was replaced with methionine, and both the first methionine and 2nd amino acid of fluorescent protein (EYFP) were deleted.

References

Bachmann, B.J. (1972) Pedigrees of some mutant strains of Escherichia coli K-12. *Bacteriol Rev* **36**: 525-557.

Datsenko, K.A., and Wanner, B.L. (2000) One-step inactivation of chromosomal genes in Escherichia coli K-12 using PCR products. *Proc Natl Acad Sci U S A* **97**: 6640-6645.

Ito, Y., Suzuki, M., and Husimi, Y. (1999) A novel mutant of green fluorescent protein with enhanced sensitivity for microanalysis at 488 nm excitation. *Biochem Biophys Res Commun* **264**: 556-560.

Lau IF, Filipe SR, Soballe B, Okstad OA, Barre FX Sherratt DJ (2003) Spatial and temporal organization of replicating *Escherichia coli* chromosomes. *Mol Microbiol* **49**: 731-743.

Mori, H., Kondo, A., Ohshima, A., Ogura, T., and Hiraga, S. (1986) Structure and function of the F plasmid genes essential for partitioning. *J Mol Biol* **192**: 1-15.

Ogura, T., and Hiraga, S. (1983) Partition mechanism of F plasmid: two plasmid gene-encoded products and a cis-acting region are involved in partition. *Cell* **32**: 351-360.

Yu, D., Ellis, H.M., Lee, E.C., Jenkins, N.A., Copeland, N.G., and Court, D.L. (2000) An efficient recombination system for chromosome engineering in Escherichia coli. *Proc Natl Acad Sci U S A* **97**: 5978-5983.

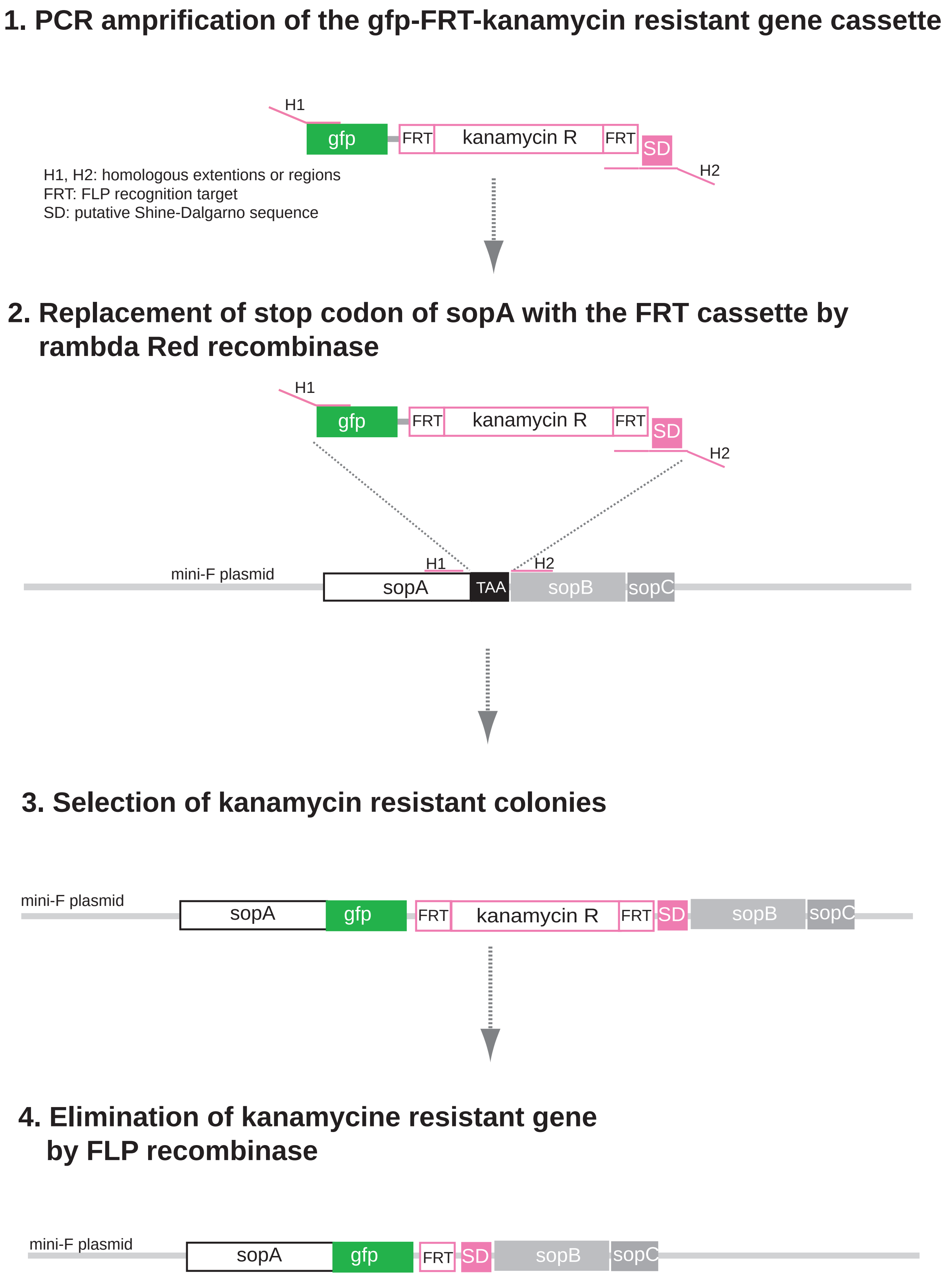
1. PCR amprification of the gfp-FRT-kanamycin resistant gene cassette
H1
gfp
FRT
kanamycin R
FRT
SD
H2
H1, H2: homologous extentions or regions
FRT: FLP recognition target
SD: putative Shine-Dalgarno sequence
2. Replacement of stop codon of sopA with the FRT cassette by rambda Red recombinase
H1
gfp
FRT
kanamycin R
FRT
SD
H2
mini-F plasmid
H1
H2
sopA
TAA
sopB
sopC
3. Selection of kanamycin resistant colonies
mini-F plasmid
sopA
gfp
FRT
kanamycin R
FRT
SD
sopB
sopC
4. Elimination of kanamycine resistant gene by FLP recombinase
mini-F plasmid
sopA
gfp
FRT
SD
sopB
sopC

Fig. S1

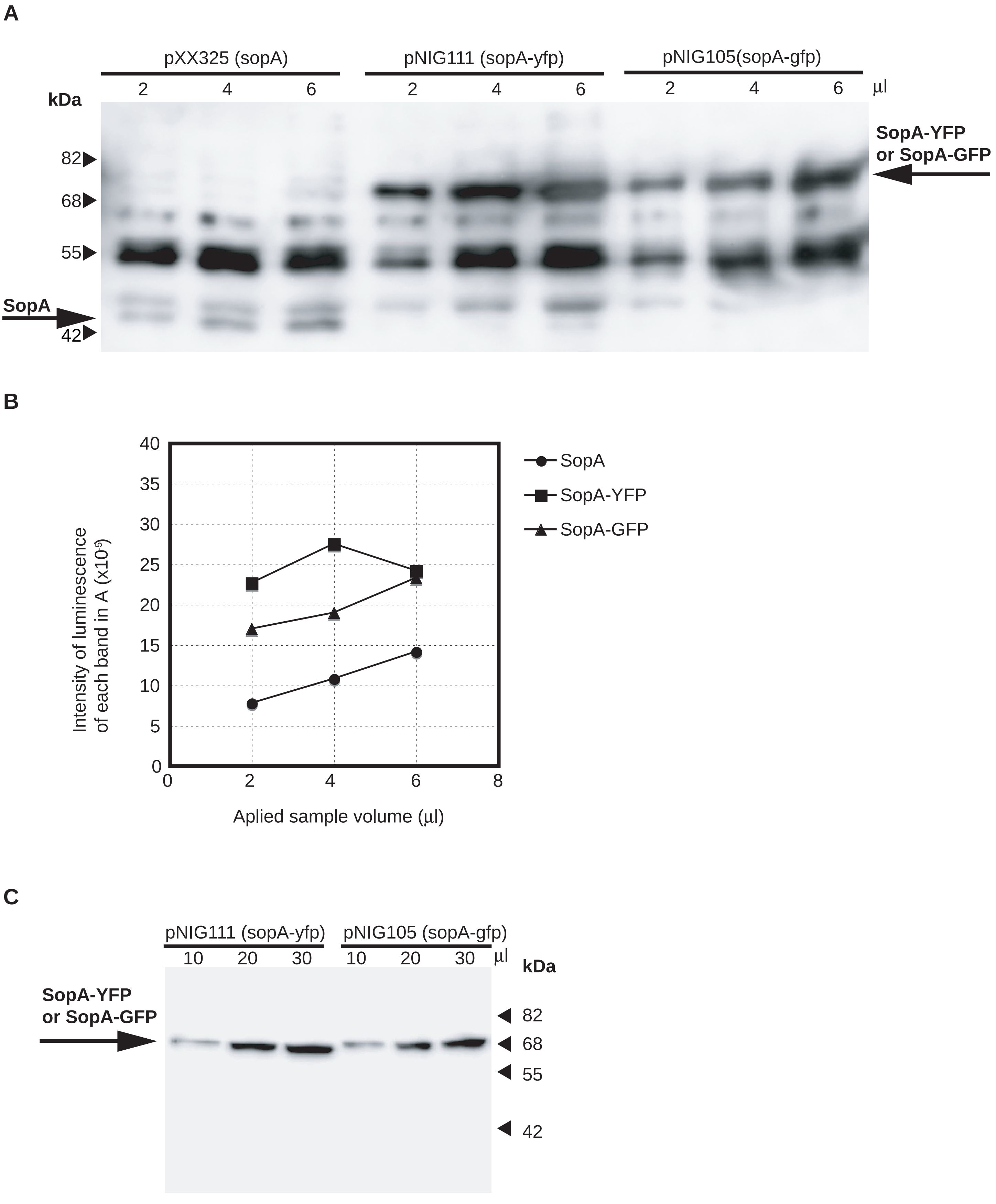

Fig. S2 Hatano and Niki

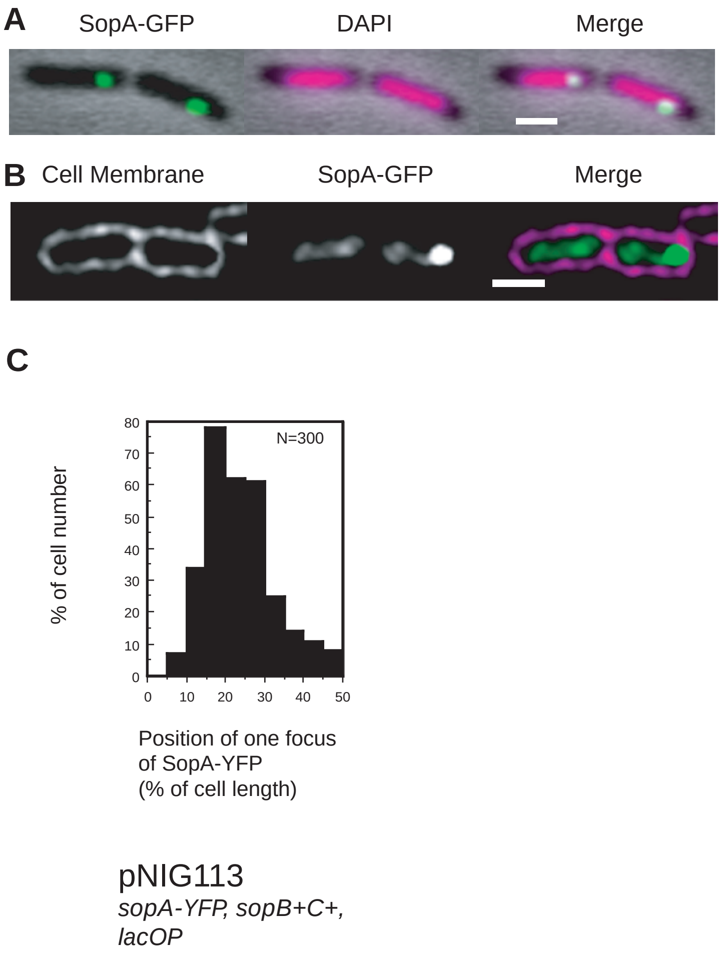

Figure S3 Hatano and Niki

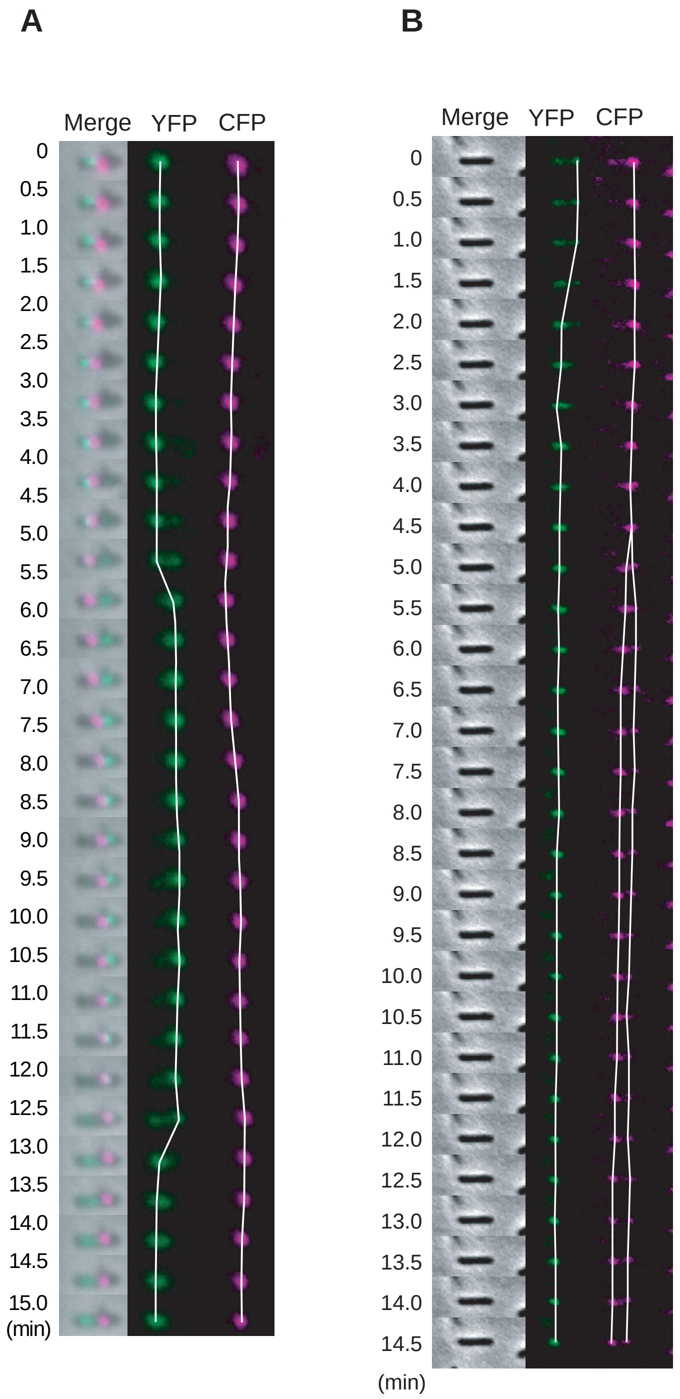

Fig. S4 Hatano and Niki
